# Obestatin inhibits apoptosis and astrogliosis of hippocampal neurons following global cerebral ischemia reperfusion via antioxidant and anti-inflammatory mechanisms

**DOI:** 10.22038/ijbms.2019.34118.8110

**Published:** 2019-06

**Authors:** Elahe Mirarab, Vida Hojati, Golamhassan Vaezi, Abdolhossein Shiravi, Mehdi Khaksari

**Affiliations:** 1 Department of Biology, Damghan Branch, Islamic Azad University, Damghan, Iran; 2 School of Medicine, Shahroud University of Medical Sciences, Shahroud, Iran

**Keywords:** Apoptosis, Astrogliosis, Brain ischemia Hippocampus, Obestatin

## Abstract

**Objective(s):**

Obestatin is a newly discovered peptide with antioxidant activities in different animal models. Recent studies have shown that Obestatin inhibits apoptosis following cardiac ischemia/reperfusion injury. Brain ischemia/reperfusion induces irreversible damage especially in the hippocampus area. This study aimed at examining the protective impact of Obestatin on apoptosis, protein expression and reactive astrogliosis level in hippocampal CA1 region of rat following transient global cerebral ischemia.

**Materials and Methods:**

Forty-eight male Wistar rats were randomly assigned into 4 groups (sham, ischemia/reperfusion, ischemia/reperfusion+ Obestatin 1, and 5 µg/kg, n=12). Ischemia induced occlusion of both common carotid arteries for 20 min. Obestatin 1 and 5 µg/kg were injected intraperitoneally at the beginning of reperfusion period and 24 and 48 hr after reperfusion. Assessment of the antioxidant enzymes and tumor necrosis factor alpha (TNF-α) was performed by ELISA method. Caspase-3 and glial fibrillary acidic protein (GFAP) proteins expression levels were evaluated by immunohistochemical staining 7 days after ischemia.

**Results:**

Based on the result of the current study, lower superoxide dismutase (SOD) and glutathione (GSH) (*P*<0.05) and higher malondialdehyde (MDA) and TNF-α levels were observed in the ischemia group than those of the sham group (*P*<0.01). Obestatin treatment could increase both SOD and GSH (*P*<0.05) and reduce MDA and TNF-α (*P*<0.05) versus the ischemia group. Moreover, obestatin could significantly decrease caspase-3 and GFAP positive cells in the CA1 region of hippocampus (*P*<0.01).

**Conclusion:**

Obestatin exerts protective effects against ischemia injury by inhibition of astrocytes activation and decreases neuronal cell apoptosis via its antioxidant properties.

## Introduction

Obestatin is a 23-amino acid peptide encoded by the ghrelin gene, originating from post-translational processing of the preproghrelin peptide. At first, it had been considered as an endogenous ligand of G-protein-coupled receptor 39 (GPR39), which counteracts with food intake induced by ghrelin and secretion of growth hormone (GH). The expression and release of obestatin in different peripheral tissues have been indicated; however, stomach and gastrointestinal tract produce obestatin like ghrelin (1, 2). 

Although the proliferation of retinal pigment epithelium cells is stimulated by obestatin (3, 4), it prevents apoptosis in pancreatic islets of both rodents and humans (5, 6).

In addition, obestatin plays a major role in the protection from myocardial ischemia and reperfusion (I/R), preconditioning ischemia, and isolated ventricular myocytes (7). A study showed anti-inflammatory and protective effects of peripheral injection of obestatin on subarachnoid hemorrhage caused by brain injury (8), while another study demonstrated that the peripheral administration of obestatin can reduce the severity of pentylenetetrazol (PTZ)-induced seizures, improve memory dysfunction, and reduce neuronal damage by limiting oxidative damage (9). 

Brain ischemia is becoming a leading cause of morbidity and mortality world-wide (10). Cerebral ischemia triggers complexe and dynamic changes, which lead to ischemic vascular injury and different degrees of the blood–brain barrier (BBB) disruption. Numerous recent studies have shown that the development of many neuroprotective treatment strategies can reduce brain damage following cerebral ischemia in animal models (11).

The hippocampal CA1 neurons are selectively vulnerable to transient global cerebral ischemia. The neuroinflammation responses that occur by immune mediators fallowing brain ischemia cause neuronal cell death. Astrocytes are the important mediators of brain that have been reported to release various pro-inflammatory factors after ischemic injury, such as intermediate filament, glial fibrilliary acid protein (GFAP) (12). Lack of GFAP protein following the middle cerebral artery occlusion is associated with increased susceptibility to ischemic brain damage in mice. Furthermore, previous evidences have shown that reactive astrocytes upregulate GFAP in many neurodegenerative conditions such as ischemia. Thus, it was extensively applied as an alternative marker of neuronal injury in brain ischemia (13). According to our literature review, despite of many studies that have been conducted on obestatin, there has been no investigation on the role of obestatin in I/R. Considering the mechanisms contributing brain ischemia as well as protective effects that have been reported about obestatin, we studied the effects of obestatin administration on neuroinflammation and caspase-3 following transient global cerebral I/R.

## Materials and Methods

Male Wistar rats, weighting 250–300 g, provided by Tehran Pasteur Institute were caged at 22-24°C, with 45-50% humidity, and 12:12 hour light/dark cycle (light on at 08:00A.M). They had free access to both food and water. The experiments were accomplished according to the Helsinki Declaration. Ethics code is IR.SHMU.REC.1397.124. Obestatin (Sigma-Aldrich Company, Germany, Hamburg) was dissolved in saline.

***Transient global cerebral ischemia model***

Transient global cerebral I/R injury model was induced using a method that was previously described, in the following groups: sham (n=12), I/R (n=12), I/R + obestatin (n=12) (1, 5 µg/kg intraperitoneally (IP, injection) in the beginning, 24, and 48 hr after reperfusion (14). Ketamine/xylazine (80 mg/kg, IP) was used to induce anesthesia in the rats and then were subjected to ischemia surgery. At first, bilateral carotid arteries were carefully separated from the sheets and vagus nerves; then, they were occluded by Yasargil Aneurism micro clips. The clips were removed for immediate reperfusion at the end of the occlusion, and blood flow restoration was visually confirmed. The rectal temperature of the rats was maintained at 36.5 ± 0.5 ^°^C during the experimental period to regulate the feedback of heating system. After surgery, animals were kept separately for 7 days in home cages with free access to food and water. 

***Experimental design and protocols***

Immunohistochemical evaluation, and determination of antioxidant and inflammatory markers were performed 7 days after inducing cerebral ischemia. The rats in Sham group, considered as controls, underwent similar procedure, except the carotid arteries occultation. After 7 days of cerebral ischemia induction, the rats were beheaded and staining was performed on half of transcardial perfusions.

***Biochemical analysis***

Seven days after induced cerebral ischemia, half of the animals in each group were anesthetized and their brains were removed for biochemical analysis. For this purpose, the hippocampal samples (6 rats in each group) were homogenized on ice in cold radioimmunoprecipitation assay (RIPA) buffer plus protease inhibitor, and then the samples were centrifuged at 3000 g for 20 min at 4 ^°^C. The supernatant was used to evaluate the enzymes (15).

***Measurement of malondialdehyde level***

Malondialdehyde (MDA) levels and lipid peroxidation index were measured in the hippocampal samples using MDA assay kit (ZellBio GmbH, Germany). The tissues were homogenized for 2 min on ice. MDA levels were measured based on the thiobarbituric acid reactive substance (TBARS) formation method. Cold 10% trichloroacetic acid (1 ml) and 10% thiobarbituric acid (2 ml) were mixed with homogenized tissues and heated at 100 ^°^C for 1 hr. After cooling of the solution and centrifuging, the precipitate was removed and pink color supernatant was moved into the microplate. Measurement of reaction mixture absorbance was performed at 535 nm by a microplate reader (ELx800, BioTek, USA). The concentration of MDA (µM) was calculated according to standard curve.

***Measurement of reduced glutathione and superoxide dismutase levels***

Reduced glutathione (GSH) and superoxide dismutase (SOD) levels were measured based on instructions of GSH and SOD assay kit (ZellBio GmbH, Germany). After adding a certain amount of phosphate-buffered saline (PBS; 100 mM, pH 7.4), the hippocampal samples were homogenized and centrifuged. The supernatants were carefully separated. Then, after GSH and SOD interacted with chromogen reagent, the absorbances were read with microplate reader (ELx800, BioTek, USA) at 412 nm and 420 nm to calculate GSH (mM) and SOD levels (U/ml).

***Measurement of tumor necrosis factor-alpha level***

The tumor necrosis factor-alpha (TNF–α) level was determined based on the TNF–α assay kit instructions (Diaclone). Wells of the microtiter strips were coated with a monoclonal antibody specific for rat TNF-α. During the first incubation, the rat TNF-α antigen and a biotinylated monoclonal antibody specific for rat TNF-α were simultaneously incubated. The enzyme (streptavidin-peroxidase) was added, after washing. After incubation and washing, a substrate solution that acts on the bound enzyme to remove the entire unbound enzyme, was added and induced a colored reaction product. The intensity of this colored product is directly proportional to the concentration of rat TNF-α present in the samples.

***Tissue preparation for staining***

Seven days after ischemia, half of animals were deeply anesthetized with ketamine using a solution containing 0.9% saline plus 4% PFA in PBS 0.1 M (pH 7.4) and transcardial perfusion was performed. Then, the brains were removed, re-fixed in the same solution for 3 days, and embedded in paraffin. Using a microtome, the coronal mass was cut into 7-µm sections according to the Paxinos atlas (3.3 to 4.2 mm posterior to bregma) in order to be stained with different methods (16).

***Immunohistochemical staining***

To identify astrocyte activation and apoptosis, immunohistochemical staining was used to evaluate caspase-3 and GFAP on 7 μm tissue sections using antibodies against caspase-3 and GFAP. After removal of paraffin by 30 min incubation at 60 ^°^C, tissue sections were cleared with xylene, and subtractive series of alcohols were applied for rehydration, and then the tissue sections were treated with 10% H_2_O_2_ in methanol for 10 min to inhibit endogenous peroxidase activity. Then, Tris buffer was used to wash the sections and after 11 min autoclaving in citrate buffer, antigen was retrieved. After washing in PBS, sections were fixed with 1% fetal bovine serum (FBS) in 0.3% TritonX-100, and then, primary antibody (Abcam, UK) with dilution ratio of 1:100 was used following an overnight incubation at 4 ^°^C. Then, to detect the antigen, goat polyclonal secondary antibody (Abcam, UK) was left for 30 min in room adding DAB (Sigma, USA). Finally, counterstaining with hematoxylin (Sigma) was performed for visualization under the microscope (17). The GFAP-positive cells were counted in the right hippocampal CA1 region (at 400×) that prepared from each slide related to each animal. The person who was in charge for counting procedures was blind to the study objectives. The number of immune positive cells was counted along a transect of 400 μm length (0.160 mm^2^) of CA1 area of the right hippocampus. The primary antibody was not included in the process of negative control slides.

***Statistical analysis***

Data were expressed as mean±SEM. The Kolmogorov–Smirnov test showed the normality of variables distribution. ANOVA was applied to compare the groups. After adjusting the level of significance, the Dunnett T3 or the Scheffe *post hoc* test was used to evaluate differences. If the variances were homogenous, the Scheffe *post hoc* test was used, and if not, Dunnett’s T3 *post hoc* test was employed. *P*≤0.05 was considered as the level of significance. Data were analyzed with SPSS Software version 16.

## Results

***Obestatin increased SOD and GSH levels after ischemia***

**Figure 1:**
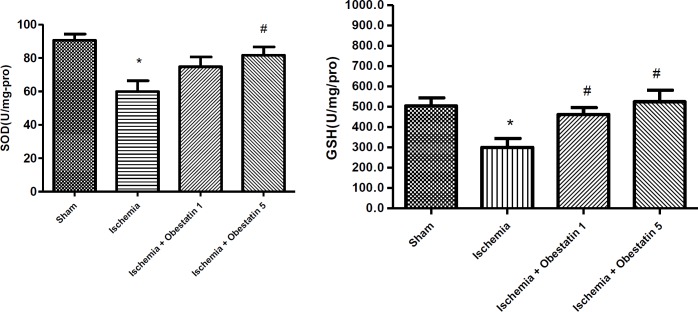
Effect of treatment with obestatin on superoxide dismutase (SOD) and reduced glutathione (GSH) concentration in the hippocampus following ischemia and reperfusion (I/R). * Significantly different compared to sham group (*P*<0.05)

There was a noticeable reduction in the SOD levels in ischemia group (59.41 U/mg-pro ± 5.33) compared to the sham (90.6 ± 6.1) group (*P*<0.05). In ischemic rats on obestatin treatment, SOD levels increased (81.34 ± 7.68) compared to the ischemic rats (*P*<0.05). GSH levels in the ischemia group (300.1 U/mg-pro ± 26.5) was decreased compared to sham (536 ± 34, *P*<0.05). In addition, in ischemic rats on obestatin treatment, the GSH levels increased (505 ± 21) compared to the ischemic rats (*P*<0.05) (Figure 1). 

***Obestatin decreased the concentration of MDA and TNF-α after ischemia***

In biochemical analyses, the MDA levels of the hippocampus in ischemic group (22.35 nmol/mg-pro ± 2.25) were higher than the sham (12.75±1.75) group (*P*<0.01). The MDA levels (14.36 µM±1.62) decreased following the treatment with obestatin compared to the ischemic group (*P*<0.05).

 The TNF-α levels of the hippocampus in ischemic group (238±8 pg/mg-pro) were higher than the sham group (159±12 pg/mg-pro) (*P*<0.01). Treatment with obestatin (5 mg/kg) decreased the TNF-α levels compared to the ischemic group (*P*<0.05) (Figure 2). 

***Obestatin reduced the activation of caspase-3 following ischemia***

The rate of active caspase-3 positive cells was significantly higher in the ischemia group (49.4% ± 4.67) compared to the sham group (5.7% ± 1.11, *P*<0.001). In the obestatin treatment group, the rate of active caspase-3 positive cells (25.2% ± 2.3) was significantly lower than ischemia group (*P*<0.01, Figure 3). 

***Obestatin reduced the level of GFAP after ischemia***

GFAP was weakly expressed in sham group (16.35% ± 1.2). Also, the rate of GFAP-positive cells was higher in ischemia group (58.4% ± 5.3) than the sham (*P*<0.001). In the obestatin treatment group, the percentage of GFAP-positive cells was lower than the ischemia group (32.5% ± 4.5, *P*<0.01) (Figure 4).

## Discussion

The current study for the first time indicated that the obestatin can significantly attenuate apoptotic cell death in CA1 pyramidal cells after brain I/R in the rat hippocampus. Moreover, our results revealed that obestatin has beneficial effect in the prevention of lipid peroxidation. Obestatin also significantly elevate antioxidant enzymes (SOD and GSH) capacity following brain ischemia. Furthermore, this study showed that obestatin attenuated TNF-α production induced by I/R. This provided the evidence that ischemia induces neuroinflammation intermediary activity of astrocytes in the hippocampal CA1 neurons. Besides, treatment with obestatin considerably reduced the expression of GFAP induced by I/R.

# Significantly different compared to ischemic group (*P*<0.05)

**Figure 2:**
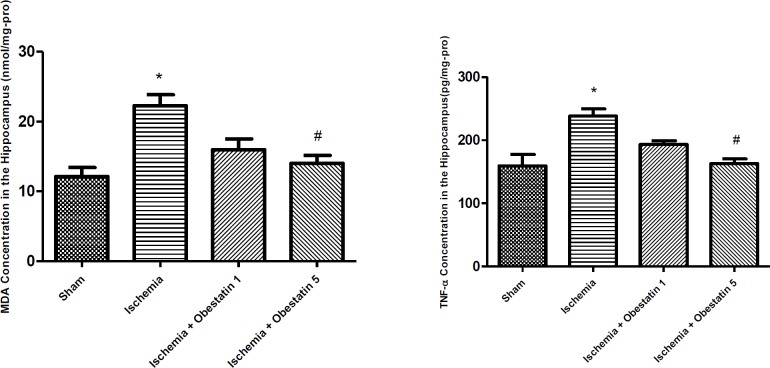
Effects of treatment with obestatin (1, 5 µg) on malondialdehyde (MDA) and tumor-necrosis factor-α (TNF-α) concentration in the hippocampus following ischemia and reperfusion (I/R), (N=6 per groups). * Significantly different compared to sham group (*P*<0.01)

# Significantly different compared to ischemic group (*P*<0.05)

**Figure 3:**
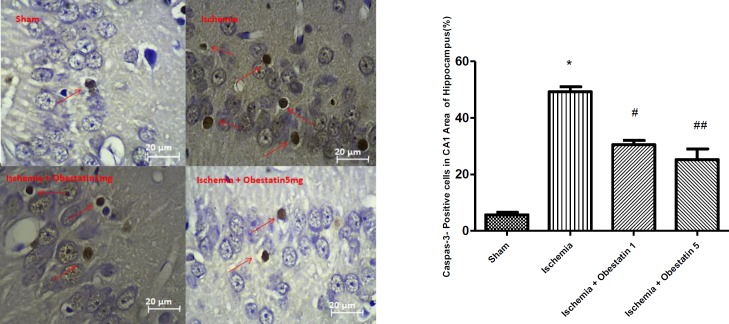
Effects of obestatin on the percentage of caspas-3 positive cells

Left: Photomicrographs of immunohistochemical staining of caspase-3 in the CA1 area of hippocampus after ischemia and reperfusion (I/R). Red arrows in the photomicrographs shows caspas-3 expression

Right: Effects of obestatin treatment on the caspase-3 levels in the hippocampal CA1 area following the cerebral ischemia. Treatment with obestatin significantly attenuated the I/R-induced increase in levels of caspase-3 expression, (N=6 per groups)

* Significantly different compared to sham group (*P*<0.001)

# Significantly different compared to ischemic group (*P*<0.01)

**Figure 4:**
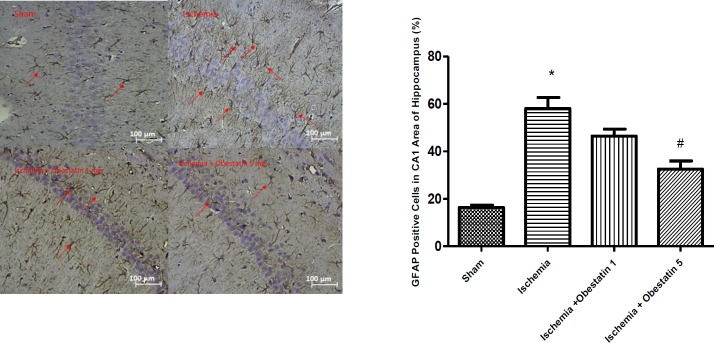
Effects of obestatin on the percentage of glial fibrillary acidic protein (GFAP)-positive cells

Left: Photomicrographs of immunohistochemical staining of GFAP in the CA1 area of hippocampus after ischemia and reperfusion (I/R). Red arrows in the photomicrographs shows GFAP expression

Right: Effects of obestatin treatment on the GFAP levels in the hippocampal CA1 area following the cerebral ischemia. Treatment with obestatin significantly attenuated the I/R-induced increase in levels of GFAP expression, (N=6 per groups)

* Significantly different compared to sham group (*P*<0.001)

# Significantly different compared to ischemic group (*P*<0.01)

The pathophysiology and mechanisms of cerebral I/Rare complex. Many studies revealed that reperfusion affects brain injury and causes a sort of processes following the restoration of blood flow such as oxidative stress, energy failure, inflammation, excitotoxicity, calcium dysregulation, activation of cell signaling pathways such as apoptosis and neuronal death (18, 19). Any factor preventing such processes is applicable to treat brain ischemia. 

Many evidences show that the reactive oxygen species (ROS) contribute to brain injuries and cerebral I/R. Blood flow reduces in brain in focal or global cerebral ischemia, while the occluded vessels should supply blood in this region. Oxygen, as a substrate for numerous enzymatic reactions, is supplied by reoxygenation or thrombolytic reperfusion in the cytosolic compartments or subcellular organelles and mitochondria. ROS are scavenged by SOD, glutathione peroxidase (GSH-Px), and catalase.

 Increased ROS and reactive nitrogen species by oxidizing proteins, damaging DNA, and increasing lipid peroxidation in cell membrane affects neuronal death (20, 21). Obestatin increases the activity of SOD and GSH-Px. Therefore, the level of DNA damage decreases, as well. MDA, a cytotoxic compound produced by lipid peroxidation, is a biomarker for oxidative stress, which indicates the production of free radical and subsequent damage in tissues (22). Antioxidant enzymes such as SOD and GSH protect cells against oxidative insult. Results of the current study indicated that SOD and GSH levels significantly reduced the plasma antioxidant activity following I/R, although in consistent with previous findings, the level of MDA significantly increased (23). However, plasma SOD and GSH significantly increased following the obestatin treatment, although the level of MDA significantly reduced in rats.

One of the possible neuroprotective mechanisms of obestatin can result from its inflammation suppression properties. Astrocytes are the versatile caretakers of the CNS. They are necessary for proper brain development, play pivotal roles in maintaining ions, neurotransmitter, water and energy homeostasis and modulate neuronal signaling (24, 25). Morphology and function of astrocytes are changed by reactive astrogliosis in many neurological disorders including neurotrauma, ischemic stroke, and neurodegenerative diseases. Reactive astrocytes also affect gene expression and GFAP upregulation (26, 27).

Moreover, based on different studies reactive astrocytes following brain ischemia play a prominent role in inflammation regulation provided by a major source of the pro-inflammatory cytokines and chemokines (28-30). The major pro-inflammatory products include monocyte chemotactic protein-1 (MCP1/CCL2), interleukin (IL) 1β, IL6, and TNF-α (31).

TNF-α is a potent pro-inflammatory cytokine. It stimulates endothelial adhesions production and inflammatory mediators release from neutrophils and microglial cells. TNF-α also enhances the permeability of the blood-brain barriers thereby contributing to secondary brain injury following cerebral ischemia. TNF-α causes direct neuronal toxicity by generating ROS via the secondary messenger ceramide to cause neuronal apoptosis (32). 

Consistent with our observation, the recent study showed that obestatin has an anti-inflammatory effect in an experimental model of colitis (33).

## Conclusion

The results of current study showed that obestatin can significantly decrease neuronal damage in CA1 area of rat hippocampus that is induced by cerebral ischemia. It seems that the neuroprotective effects of obestatin may arise from many mechanisms such as inhibition of apoptosis and asterogeliosis and also improve the antioxidant system. If the protective effects of obestatin on pathological conditions are taken into consideration, this peptide can be introduced as a therapeutic agent for various diseases, such as cerebral I/R; however, further researches are highly suggested to more identify the cellular mechanisms in this realm. 
